# Conformational Transitions of the Pituitary Adenylate Cyclase-Activating Polypeptide Receptor, a Human Class B GPCR

**DOI:** 10.1038/s41598-017-05815-x

**Published:** 2017-07-14

**Authors:** Chenyi Liao, Xiaochuan Zhao, Matthias Brewer, Victor May, Jianing Li

**Affiliations:** 1 Department of Chemistry, University of Vermont, Burlington, VT 05405 USA; 2 Department of Neurological Sciences, College of Medicine, University of Vermont, Burlington, VT 05405 USA

## Abstract

The G protein-coupled pituitary adenylate cyclase-activating polypeptide receptor (PAC1R) is a potential therapeutic target for endocrine, metabolic and stress-related disorders. However, many questions regarding the protein structure and dynamics of PAC1R remain largely unanswered. Using microsecond-long simulations, we examined the open and closed PAC1R conformations interconnected within an ensemble of transitional states. The open-to-closed transition can be initiated by “unzipping” the extracellular domain and the transmembrane domain, mediated by a unique segment within the β3-β4 loop. Transitions between different conformational states range between microseconds to milliseconds, which clearly implicate allosteric effects propagating from the extracellular face of the receptor to the intracellular G protein-binding site. Such allosteric dynamics provides structural and mechanistic insights for the activation and modulation of PAC1R and related class B receptors.

## Introduction

The heptahelical GPCR family consists of over 850 members critical to the homeostatic control of all physiological processes. With their wide distribution and diverse activities, GPCRs are major targets of current phamaceuticals^1^. Distinct from widely studied class A members^2–7^, class B GPCRs are far less understood. They possess a large extracellular domain (ECD) in addition to the typical heptahelical transmembrane (7TM) structure in all GPCRs. Using available structural information, we have constructed full-length models of a class B GPCR — PAC1R (*ADCYAP1R1*) — for microsecond-long molecular dynamics (MD) simulations. PAC1R signalling triggered by a bioactive peptide, PACAP, has gained much interest in physiological and behavioral neuroscience because of its association with chronic stress-related psychopathologies, including posttraumatic stress disorder and chronic pain^8, 9^. These are obvious global health issues and yet for reasons still unclear, no small-molecule agonists or antagonists to PAC1R are identified for potential therapeutics, despite many large-scale compound interrogation campaigns by the pharmaceutical industry. There are many isoforms of PAC1R depending on the absence and/or presence of two 84 base pair Hip (exon 14) and Hop (exon 15) cassettes and the 63 base pairs (exons 4–6, 21 amino acids) within the β3 and β4 strands of the ECD^10^. In this work, we employed computational technology to model the most physiologically relevant brain isoform: the PAC1null receptor with the 21-amino-acid (21-aa) ECD insert^8, 9^.

Although the PAC1R ECD structure has been determined experimentally^11, 12^, the 7TM structure as well as the relative position and orientation of the ECD remains unknown. In a previous PAC1R modeling study^13^, the full-length PAC1R was proposed with an outstretched ECD that appears at odds with recent evidence from electron microscopy and computer simulations of the glucagon receptor (GCGR)^14^. Given recent success of MD studies of GPCRs^4–6, 14, 15^, especially those on the microsecond scale, we attempted to elucidate the molecular mechanism underlying PAC1R conformational transitions. The resulting ensemble of receptor states yielded unsuspected results that should provide important insights to signalling mechanisms and potential druggable molecular target sites.

## Results

**Figure 1:**
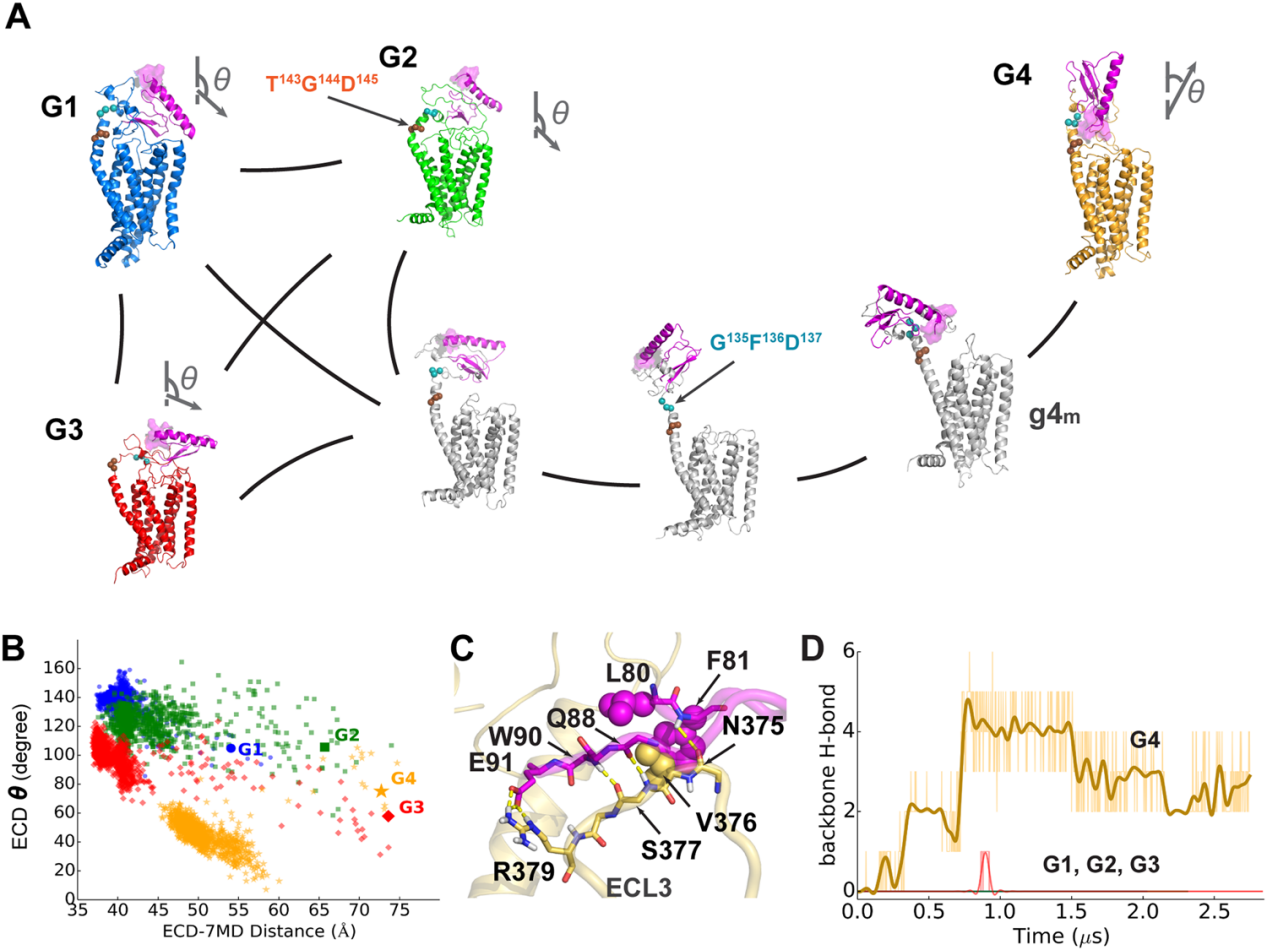
The open (G4) and closed (G1-G3) states of PAC1R. (**A**) Diagram of the PAC1R conformational transition from the MSM and transition-path theory. Vectors (N-to-C of helix 1 in ECD) show the ECD orientations, and the ECD N-term is highlighted with a purple surface. (**B**) Plots of the ECD tilt angle (*θ*) against the ECD-7TM distance. Starting points are labelled with larger markers. (**C**) The “zipper” between res. 80–91 and ECL3 in the open state. Key polar and hydrophobic side chains are shown in stick and sphere respectively. (**D**) Time evolution of the number of H-bonds defined in (**C**). The curves of G1-G3 mostly overlap. The styles of all figures in this work are consistent with Fig. 1.

Using the Markov state model (MSM)^16^ and transition-path theory^17^, we constructed the MSM transition matrix between the open and closed conformations — to describe millisecond-scale dynamics toward the timescale of GPCR activation. According to the shortest transition pathways, there are relatively short conversion paths among the closed states (G1, G2, and G3) but the open state (G4) is rather remote (see Fig. 1A and Supplementary Table 2), suggesting some partition in the conformational states. Consistently, the simulations of G1-G3 revealed conformations of similar ECD tilt angles of 100–150 degrees (see Fig. 1B and Supplementary Fig. 6) with the N-terminal helix angled toward the 7TM (the “closed” state); the conformations from the simulation of G4 exhibit ECD tilt angles of 20–60 degrees, when the ECD center is relatively distant from the 7TM (the “open” state).

Regarding the minimum transition flux, the conversion among the closed states (G1, G2, and G3) is on the order of ten microseconds, while the transition from the open states (G4) to a closed one is estimated to require over several hundred microseconds (see Supplementary Table 2). For a rational explanation of the distinct time scales, we examined the conformational changes along these transition pathways. First, throughout the course of the simulations, the ECDs and 7TMs maintain their overall folds for microseconds, as indicated by the backbone RMSDs near 1 and 3 Å, respectively (see Supplementary Fig. 7). These contrasted with the relative mobility of the intervening flexible linker region, which adopted a wide range of conformations critical to the open and closed transitions. For conversions within closed states, the rate-limiting step mainly involves a helix-coil transition at the linker (see Fig. 1A). The ECD can rotate to different extents with the linker being mostly helical into G1-like states, the linker forming a T^143^G^144^D^145^ kink into G2-like states, or even the entire linker unwinding into G3-like states. Indeed, the microsecond-long timescale of transitions between the closed states is comparable to that for helical nucleation (20–70 µs)^18^. However, the open-to-closed transition on the near-millisecond timescale requires cooperative changes at the extracellular face of the receptor, especially interactions between the β3-β4 loop of the ECD and the extracellular loop 3 (ECL3) of the 7TM, as well as a subsequent rotation of the ECD. Our analyses suggest that the transition is initiated by the separation of the β3-β4 loop from ECL3 (see Fig. 1C and g4_m_ of Fig. 1A).

The long-lasting open conformations of PAC1R resemble the GCGR open-state model^14^ with respect to the ECD orientation and the relative position to the 7TM. However, there is a notable difference between these two GPCRs in the open-state. The open state of GCGR is stabilized upon binding to glucagon, but converts to a closed state within 0.3 μs upon glucagon removal^14^. However, our open-state PAC1R model is stable in the absence of any ligand. Such stability is exclusively attributed to the backbone zipper interactions between the β3-β4 loop and ECL3 (see Fig. 1C). The open state forms abundant contacts between ECD and ECL3, which are mostly from res. 80–91 within the first half of the β3-β4 loop (see Supplementary Fig. 10). Major contacts are backbone hydrogen bonds (H-bonds, *i.e*. F81-N375^ECL3^ and Q88-S377^ECL3^-W90), a side-chain salt bridge (E91-R379^ECL3^), and an aromatic cluster (L80-F81-V376^ECL3^), which together stabilize the ECD in the open state. The zipper represents a singular feature of the stable open conformations that is absent in all closed states. Notably, this is unique to PAC1R and not found in the related receptors (VPAC1/2R), or even GCGR or CRF1R.

When we removed the 21-aa sequence from our final models and tested the consequence of lost ECD-ECL3 contacts with MD simulations, the open-state model showed a rapid transition toward a G3-like closed state within 0.1 μs (see Supplementary Fig. 8) — in line with that observed for GCGR^14^, which lacks a comparable segment in the β3-β4 loop. Thus, the 21-aa insert into the β3-β4 loop is crucial to sustain the open-state conformations, the dynamics of which may explain many unique features of PAC1R signaling.

**Figure 2:**
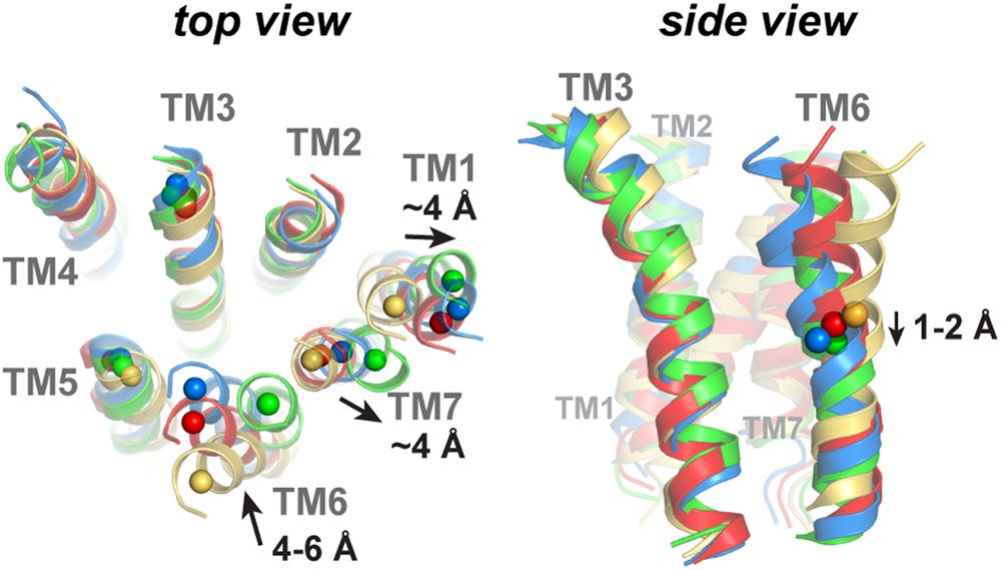
Top and side views of the final models to illustrate the helical rearrangements during the open-to-closed transition. *Left:* the center-of-mass (COM) of the first four residues in a helical turn of the TM helix. *Right:* the COMs of TM6 are represented as spheres to guide the eye.

During the open-to-closed transition, multi-step conformational changes were found to follow the β3-β4 loop “unzipping” from ECL3 (see g4_m_ of Fig. 1A). A 100-degree rotation of the ECD is first enabled, via changes in the G^135^F^136^D^137^ coil of the linker (see Fig. 1A). As PAC1R alters the ECD orientation, helical rearrangements occur concurrently. Before “unzipping”, the movement of TM6 and TM7 are constrained by ECL3. But once ECL3 is released from the β3-β4 loop, TM6 and TM7 become flexible. Concurrently, TM1 migrates outward for over 4 Å with respect to TM3; the same outward migration also occurs for the adjacent TM7. To adjust to the change of TM7 and ECL3, TM6 must slightly dip for 2 Å and migrate closer by 4–6 Å to TM3 (see Fig. 2 and Supplementary Fig. 9). The significant changes in TM6 agree with a recent study of GCGR^19^ which an antagonist restrains the TM6 dynamics. In short, concerted changes are suggested during PAC1R open-to-closed transition, including rearrangements of TM1, TM6, and TM7.

**Figure 3:**
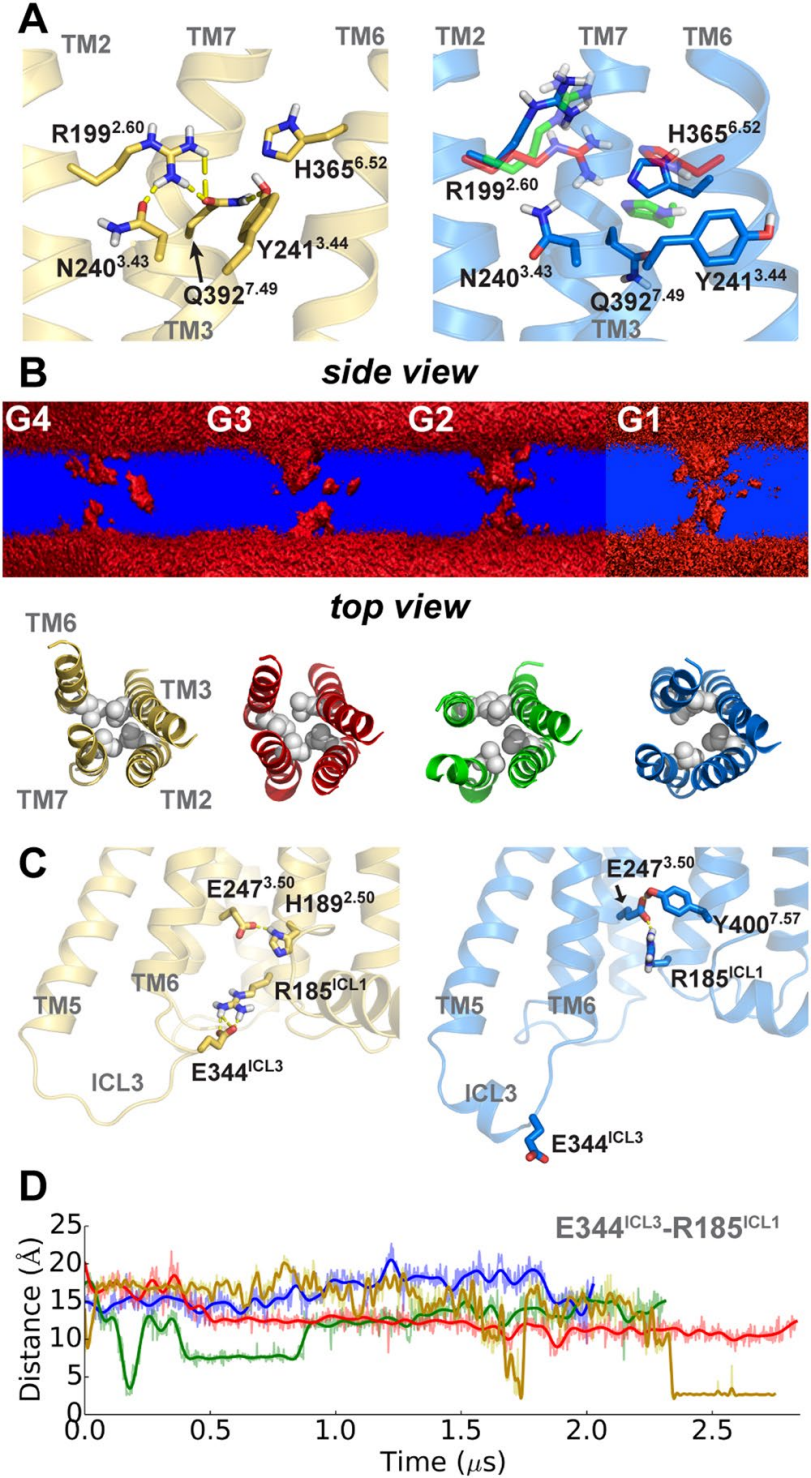
Interactions within the 7TM. (**A**) A H-bond network within the 7TM. (**B**) Water-density cross section with the hydrophobic region of L192^2.53^, L244^3.47^, L358^6.45^, and V396^7.53^ (spheres). A penetration is suggested in G1 and G2. (**C**) The salt bridge E344^ICL3^-R185^ICL1^ in two different states. (**D**) Time evolution of nearest polar atom distance in E344^ICL3^-R185^ICL1^.

As PAC1R changes toward the close state, the helical rearrangements necessarily result in a reshuffling of interaction networks within the entire 7TM domain. Displacements of TM6 and TM7 (see Supplementary Fig. 10) with respect to TM3 have direct impacts on their polar contact networks. In the open state, the H-bond network in the extracellular cavity features N240^3.43^-R199^2.60^-Q392^7.49^-Y241^3.44^ (Wootten numbering^20^, Fig. 3A) involving TM2, TM3, and TM7. During the open-to-closed transition, the inward shift of TM6 allows H365^6.52^ to join the H-bond network as observed in the simulation of G3. However, as PAC1R proceeds to other closed states like G1 or G2 with an increase in TM3-TM7 separation, and as TM6 migrates inward and downward, the H-bond network within the receptor is disrupted and eventually vanishes (see Fig. 3A). Consequently, without constraints from the H-bond network, the side chains of residues such as R199^2.60^ become mobile. These observations are consistent with prior studies of the VPAC1 receptor^21^ (60% sequence identity with PAC1R in 7TM) in which N240^3.43^, R199^2.60^, and Q392^7.49^ are crucial for the receptor activation. Alongside, the water-density cross section in the 7TM was also found altered during the open-to-closed transition. The volume of the extracellular cavity enlarges gradually from G4 to G1, mainly due to the loss of the persistent H-bond network and the hydrophobic packing of L192^2.53^, L244^3.47^, L358^6.45^, and V396^7.53^ (see Fig. 3B). In addition, the intracellular cavity near the G protein-binding site was also found to slightly expand from G4 to G1. For the first time in class B GPCRs, an intrinsic water pathway was observed in the G1 and G2 states (see Fig. 3B), which may be involved in the switching of PAC1R downstream signaling.

In the intracellular face of PAC1R, a salt bridge (E344^ICL3^-R185^ICL1^) and a H-bond (E247^3.50^-H189^2.50^) of the open state distinguish it from the closed states (see Fig. 3C). The salt bridge, absent from the original model, is steadily formed after 2.3 µs (see Fig. 3D). Given such restraint, the opening of the intracellular cavity is still limited. Instead, R185^ICL1^ in all the closed states is associated with E247^3.50^ and Y400^7.57^. However, none of our PAC1R conformations appears to have the outward movement of TM6 intracellular end as in the active structures of human class A GPCRs^3^. Hence without the presence of an agonist, PAC1R does not exhibit opening of the G protein-binding site^19, 22^ in any conformational state observed.

**Figure 4:**
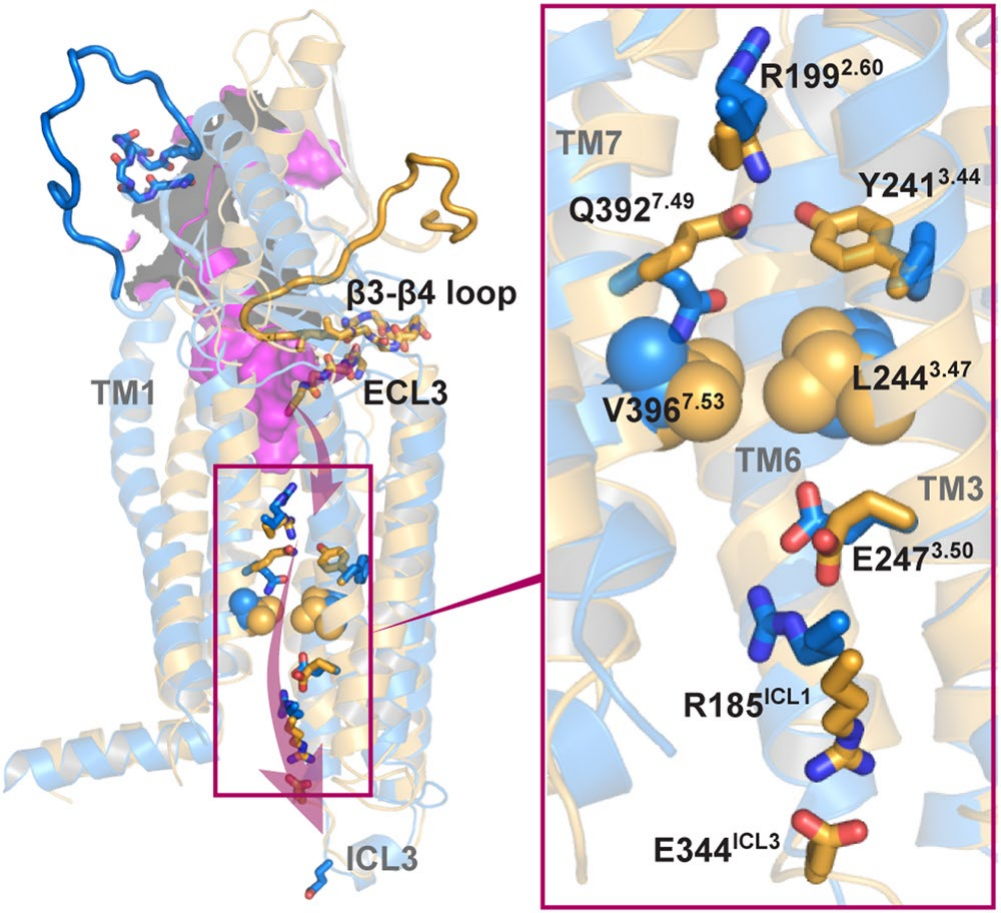
The allosteric effect shown in the open-to-closed transition of PAC1R, which is likely involved in PACAP-induced activation. TM2 is hidden for clarity. The ECD-PACAP structure (PDB ID: 2JOD, purple) is overlapped with a closed-state model.

In summary, using state-of-the-art modeling and simulation technology, we describe the conformational features of PAC1R during the open-to-closed transition, which provides insights into the ligand-binding and activation of PAC1R. The allosteric effects are clear from conformational changes that propagate from the ECD to the 7TM intracellular face (see Fig. 4). Our findings may offer strategies to inhibit class B GPCRs by restraining the allosteric dynamics. Given the alternate polar and hydrophobic layers in the 7TM, PAC1R likely has a distinct activation mechanism from class A GPCRs^4, 5^ in which the 7TMs are largely hydrophobic from allosteric data. However, to connect the conformational changes from ECD through 7TM, PACAP interactions in the extracellular cavity may be essential. This leads to our hypothesis that PACAP binds to the PAC1R in the open state to facilitate receptor transitions to a closed state (see Supplementary Fig. 11). The finding of such transitions can be crucial for PAC1R-related class B GPCRs which can be examined with mechanistic detail once the molecular tools and reagents for the PAC1 receptor become available.

## Methods

### Model Preparation

Our homology models of the human PAC1null receptor (res. 26–421) were built with the ECD template from the crystal structure (PDB ID: 3N94)^11^ and the 7TM templates from two related class B receptors, GCGR (PDB ID: 4L6R)^23^ and the corticotropin-releasing factor receptor 1 (CRF1R, PDB ID: 4K5Y)^22^, which share 41% and 31% sequence identity with PAC1R in the 7TM respectively. As the PAC1null receptor with the 21-aa ECD insert is predominant in brain, the 21-aa sequence between the β3 and β4 strands (res. 89–109, which are omitted from the ECD template) was first modeled as an extended loop. The linker (res. 126–149) was primarily helical with a coiled N-terminal. We built four starting homology models (each of which underwent a multiple-step refinement with a 100-ns MD simulation), and gained two structurally stable models with similar 7TM structures but distinct ECD orientations. Next, we used one stable model from GCGR template (with a higher sequence identity) to generate a few models differing in ECD orientations by rotating selective backbone dihedrals in the linker. Those models were simulated in the membrane for over 20~50 ns MD simulations, leading to models of four major ECD orientation partitions (G1, G2, G3, and G4) for further production runs (see Supplementary Information A). To mimic the membrane-bound environment, the 7TM of each protein model was embedded in the bilayer model of 1-palmitoyl-2-oleoyl-sn-glycero-3-phosphocholine (POPC), while the ECD was fully exposed to the solvent. We used the membrane builder of CHARMM-GUI^24^ to build the protein/membrane complex systems^25^, each of which contains a PAC1R model, a lipid bilayer of ~219 POPC molecules, ~28,500 TIP3P water molecules, counter ions, and 0.15 M NaCl, totaling near 126,000 atoms in a periodic box ~95 × 95 × 134 Å^3^.

### Simulation Setup

All simulations were performed with the CHARMM36-cmap force field^26^, an improved model for the modeling and simulation studies of proteins. Energy minimization and equilibrations of 20~50 ns each were performed using the NAMD package^27^. MD productions of 2~2.9 µs for G1, G2, G3, and G4 were carried out on the specialized Anton supercomputer using the Anton software 2.13.0^28^. Both equilibration and production runs were performed in the NPT ensemble (310 K, 1 bar, Berendesn thermostat and semi-isotropic barostat) with a time step of 2 fs. The particle mesh Ewald (PME) technique was used for the electrostatic calculations. The van der Waals and short-range electrostatics were cut off at 12.0 Å with switch at 10.0 Å.

### Data Analysis

Our data analysis includes conformational analysis, orientation and distance analysis, water dynamics, and conformational dynamics along the transition pathway. Conformation, orientation, and distance analyses were performed with TCL scripts implemented in VMD 1.9.1^29^ and plotted by matplotlib^30, 31^. Polar contacts within 3.6 Å were shown by Pymol (Schrödinger, LLC). RMSDs of the ECD core (res. 30–77 and res. 112–125) and the 7TM were computed by backbone alignments on the ECD crystal structure (PDB ID: 3N94) and the crystal structures of GCGR (PDB ID: 4L6R and 5EE7) and CRF1R (PDB ID: 4K5Y), respectively. The tilt angle of ECD is defined as the angle between the vector along the N-terminal helix and the Z-axis. The vector along the N-terminal helix is calculated by summing the C = >O vectors along the helical res. 30–47. ECD-7TM separations were calculated by the center-of-mass (COM) distance between the ECD core and 7TM. The extracellular end separation between TM helices was measured based on the COM of the first four residues of each helix at the extracellular side. TM6 (res. 351–371) shift along the z direction was calculated by Z-COM difference with TM3 relatively fixed. Water dynamics along the 7TM were analyzed by gridcount^32^, an analysis tool for Gromacs^33^. Format conversions were conducted by VMD^29^. Each microsecond trajectory of the last 100 ns was used and aligned to the 7TM backbone for the water density calculations.

To construct the transition pathways between the open and closed conformations, we used the MSMBuilder 3.2.0^34, 35^ program to build a reversible MSM, from which the shortest transition pathways were computed based on the transition-path theory^17, 36–38^ (see Supplementary Information B). In MSM, the vector of probabilities of the system to be in any of its state *m* at time (*nτ*) are given by the *Chapman-Kolmogorov equation* ^16, 39^

1 $${\bf{p}}(n\tau )={\bf{p}}(0){\bf{T}}(n\tau )={\bf{p}}(0){[{\bf{T}}(\tau )]}^{n}$$

where *τ* denotes the observation interval (or lag time), **T**(*τ*) denotes a *m* × *m* transition matrix that contains element *T*_*ij*_, the probability of going from state *i* to state *j* within time *τ*. The rate of observed *A* → *B* transitions per time unit *τ* is given by:

2 $${k}_{AB}=F/(\tau \sum _{i=1}^{m}{\pi }_{i}(1-{q}_{i}^{+}))$$

where *F* is the total transit flux, *π*_*i*_ is the Boltzmann distribution for equilibrium MD, and ${q}_{i}^{+}\,$is defined as the probability of the system at state *i* will leave *A* and continue to *B* ^17, 36, 40, 41^.

## Electronic supplementary material

Supplementary Information

## References

[1] Lappano R, Maggiolini M (2011). G protein-coupled receptors: novel targets for drug discovery in cancer. Nat. Rev. Drug Discovery.

[2] Millar RP, Newton CL (2010). The year in G protein-coupled receptor research. Mol. Endocrinol..

[3] Lebon G (2011). Agonist-bound adenosine A(2A) receptor structures reveal common features of GPCR activation. Nature.

[4] Li JN, Jonsson AL, Beuming T, Shelley JC, Voth GA (2013). Ligand-Dependent Activation and Deactivation of the Human Adenosine A(2A) Receptor. J. Am. Chem. Soc..

[5] Dror RO (2011). Activation mechanism of the beta(2)-adrenergic receptor. Proc. Natl. Acad. Sci. USA..

[6] Yuan SG, Hu ZQ, Filipek S, Vogel H (2015). W246(6.48) Opens a Gate for a Continuous Intrinsic Water Pathway during Activation of the Adenosine A(2A) Receptor. Angew. Chem. Int. Ed..

[7] Liao C (2017). Capturing the multiscale dynamics of membrane protein complexes with all-atom, mixed-resolution, and coarse-grained models. Phys. Chem. Chem. Phys..

[8] Harmar AJ (2012). Pharmacology and functions of receptors for vasoactive intestinal peptide and pituitary adenylate cyclase-activating polypeptide: IUPHAR Review 1. Brit J Pharmacol.

[9] Vaudry D (2009). Pituitary Adenylate Cyclase-Activating Polypeptide and Its Receptors: 20 Years after the Discovery. Pharmacol. Rev..

[10] Blechman J, Levkowitz G (2013). Alternative Splicing of the Pituitary Adenylate Cyclase-Activating Polypeptide Receptor PAC1: Mechanisms of Fine Tuning of Brain Activity. Front. Endocrinol. (Lausanne).

[11] Kumar S, Pioszak A, Zhang C, Swaminathan K, Xu HE (2011). Crystal Structure of the PAC1R Extracellular Domain Unifies a Consensus Fold for Hormone Recognition by Class B G-Protein Coupled Receptors. PLoS ONE.

[12] Sun CH (2007). Solution structure and mutational analysis of pituitary adenylate cyclase-activating polypeptide binding to the extracellular domain of PAC1-Rs. Proc. Natl. Acad. Sci. USA..

[13] Wu L, Guang W, Chen X, Hong A (2014). Homology modeling and molecular docking of human pituitary adenylate cyclase-activating polypeptide I receptor. Mol. Med. Rep..

[14] Yang L (2015). Conformational states of the full-length glucagon receptor. Nat. Commun..

[15] Yuan S (2016). The Molecular Mechanism of P2Y1 Receptor Activation. Angew. Chem. Int. Ed. Engl..

[16] Prinz JH (2011). Markov models of molecular kinetics: Generation and validation. J. Chem. Phys..

[17] Weinan E, Vanden-Eijnden E (2006). Towards a theory of transition paths. J. Stat. Phys.

[18] De Sancho D, Best RB (2011). What Is the Time Scale for α-Helix Nucleation?. J. Am. Chem. Soc..

[19] Jazayeri A (2016). Extra-helical binding site of a glucagon receptor antagonist. Nature.

[20] Wootten D, Simms J, Miller LJ, Christopoulos A, Sexton PM (2013). Polar transmembrane interactions drive formation of ligand-specific and signal pathway-biased family B G protein-coupled receptor conformations. Proc. Natl. Acad. Sci. USA..

[21] Chugunov AO (2010). Evidence that interaction between conserved residues in transmembrane helices 2, 3, and 7 are crucial for human VPAC1 receptor activation. Mol. Pharmacol..

[22] Hollenstein K (2013). Structure of class B GPCR corticotropin-releasing factor receptor 1. Nature.

[23] Siu FY (2013). Structure of the human glucagon class B G-protein-coupled receptor. Nature.

[24] Jo S, Kim T, Iyer VG, Im W (2008). CHARMM-GUI: a web-based graphical user interface for CHARMM. J. Comput. Chem..

[25] Liao C (2016). Conformational Heterogeneity of Bax Helix 9 Dimer for Apoptotic Pore Formation. Sci. Rep.

[26] Best RB (2012). Optimization of the Additive CHARMM All-Atom Protein Force Field Targeting Improved Sampling of the Backbone φ, ψ and Side-Chain χ1 and χ2 Dihedral Angles. J. Chem. Theory Comput..

[27] Phillips JC (2005). Scalable molecular dynamics with NAMD. J. Comput. Chem..

[28] Shaw, D. E. *et al*. In Proceedings of the Conference on High Performance Computing Networking, Storage and Analysis 1–11 (ACM, Portland, Oregon, 2009).

[29] Humphrey W, Dalke A, Schulten K (1996). VMD: visual molecular dynamics. J. Mol. Graph. Model..

[30] Hunter JD (2007). Matplotlib: A 2D graphics environment. Computing in Science & Engineering.

[31] Liao C (2015). Melittin aggregation in aqueous solutions: insight from molecular dynamics simulations. J. Phys. Chem. B.

[32] Beckstein O, Sansom MSP (2003). Liquid-vapor oscillations of water in hydrophobic nanopores. Proc. Natl. Acad. Sci. USA..

[33] Berendsen HJC, Vanderspoel D, Vandrunen R (1995). Gromacs - a Message-Passing Parallel Molecular-Dynamics Implementation. Comput. Phys. Commun..

[34] Bowman GR, Huang XH, Pande VS (2009). Using generalized ensemble simulations and Markov state models to identify conformational states. Methods.

[35] Bowman GR, Beauchamp KA, Boxer G, Pande VS (2009). Progress and challenges in the automated construction of Markov state models for full protein systems. J. Chem. Phys..

[36] Noe F, Schutte C, Vanden-Eijnden E, Reich L, Weikl TR (2009). Constructing the equilibrium ensemble of folding pathways from short off-equilibrium simulations. Proc. Natl. Acad. Sci. USA..

[37] Berezhkovskii A, Hummer G, Szabo A (2009). Reactive flux and folding pathways in network models of coarse-grained protein dynamics. J. Chem. Phys..

[38] Metzner P, Schutte C, Vanden-Eijnden E (2009). Transition Path Theory for Markov Jump Processes. Multiscale Modeling & Simulation.

[39] Chodera JD, Singhal N, Pande VS, Dill KA, Swope WC (2007). Automatic discovery of metastable states for the construction of Markov models of macromolecular conformational dynamics. J. Chem. Phys..

[40] Du R, Pande VS, Grosberg AY, Tanaka T, Shakhnovich ES (1998). On the transition coordinate for protein folding. J. Chem. Phys..

[41] Bolhuis PG, Chandler D, Dellago C, Geissler PL (2002). Transition path sampling: throwing ropes over rough mountain passes, in the dark. Annu. Rev. Phys. Chem..

