# Supplementary material for: Conformational Transitions of the Pituitary Adenylate Cyclase-Activating Polypeptide Receptor, a Human Class B GPCR
Source: Sci Rep. 2017 Jul 14;7:5427. doi: 10.1038/s41598-017-05815-x (PMC5511175; doi:10.1038/s41598-017-05815-x)
Supplement: Supplementary file 1 — Supplementary Information [file 41598_2017_5815_MOESM1_ESM.pdf]

Supplementary Information

Conformational Transitions of the Pituitary Adenylate Cyclase-Activating Polypeptide Receptor, a Human Class B GPCR

Chenyi Liao[1], Xiaochuan Zhao[1], Matthias Brewer[1], Victor May[2], and Jianing Li [1,*]

[1] Department of Chemistry, University of Vermont, Burlington, VT 05405, USA

[2] Department of Neurological Sciences, College of Medicine, University of Vermont, Burlington, VT 05405, USA

* Corresponding Author:
Jianing Li (jianing.li@uvm.edu)
Department of Chemistry, University of Vermont, Burlington, VT 05405, USA

Table of Contents

Supplementary Information A: The initial model preparations, the Wootten numbering, and the gridcount method

Supplementary Information B: The construction, validation, and analyses of our Markov state model (MSM)

Supplementary Information C: Details of the PAC1R orientations and conformations.

Supplementary Information D: The helical distance and the residue-residue minimum-distance maps

Supplementary Information E: Our proposed PACAP-bound models in the open and closed states

Supplementary Information A: The initial model preparations, the Wootten numbering, and the gridcount method

The initial model preparation. Amino acid sequence analysis indicates that the 7TM is conserved with 41% identity between the GCGR and PAC1R, and 31% between the CRF1R and PAC1R. We first generated several full-length 3D atomistic models using SWISS-MODEL,[1] using both GCGR (PDB code: 4L6R)[2] and CRF1R (PDB code: 4K5Y)[3] as the 7TM templates. The sequence alignments are displayed in Supplementary Table 1. The target ECD used the crystal structure (PDB code: 3N94)[4] as the template. The full-length homology models in membrane-bound environment went through a multiple-step refinement (energy minimization and equilibration) with 100-ns MD simulation. We obtained two structurally stable models converged to similar 7TM structures but different ECD orientations: one 7TM template is from GCGR, the other is from CRF1R. (Supplementary Fig. 1) *Despite the initial full-length models, the critical ECD orientations remain to be understood, especially for further study of ligand binding*. As PACAP and glucagon belong to the same peptide family, we chose the stable homology model with 7TM from GCGR template to generate a few models differing in ECD orientations by rotating the backbone dihedrals in the linker (Supplementary Fig. 2). We relaxed each model with major stages of energy minimization, 200-ps equilibration, and 20~50-ns short simulations. Four models of different ECD orientations were then selected (labeled as G1, G2, G3, and G4) for further simulations.

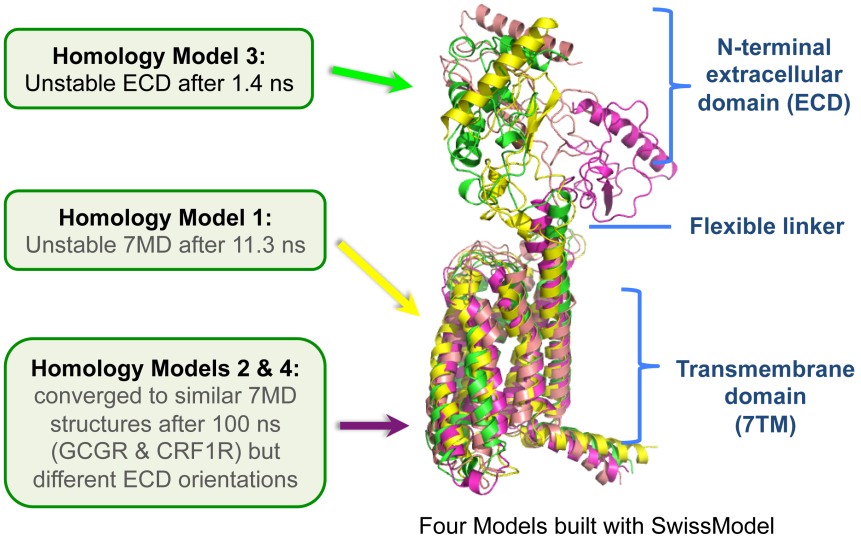

Supplementary Fig. 1 PAC1R homology modeling and refinements. Homology models 2 and 4 (pink and magenta) converged to similar TMD structures after 100 ns (GCGR and CRF1R) but different ECD orientations. Homology models 1 and 3 (yellow and green) are unstable (with visible conformational distortions) after 1.4 and 11.3 ns. Model 2 was selected for microsecond simulations.

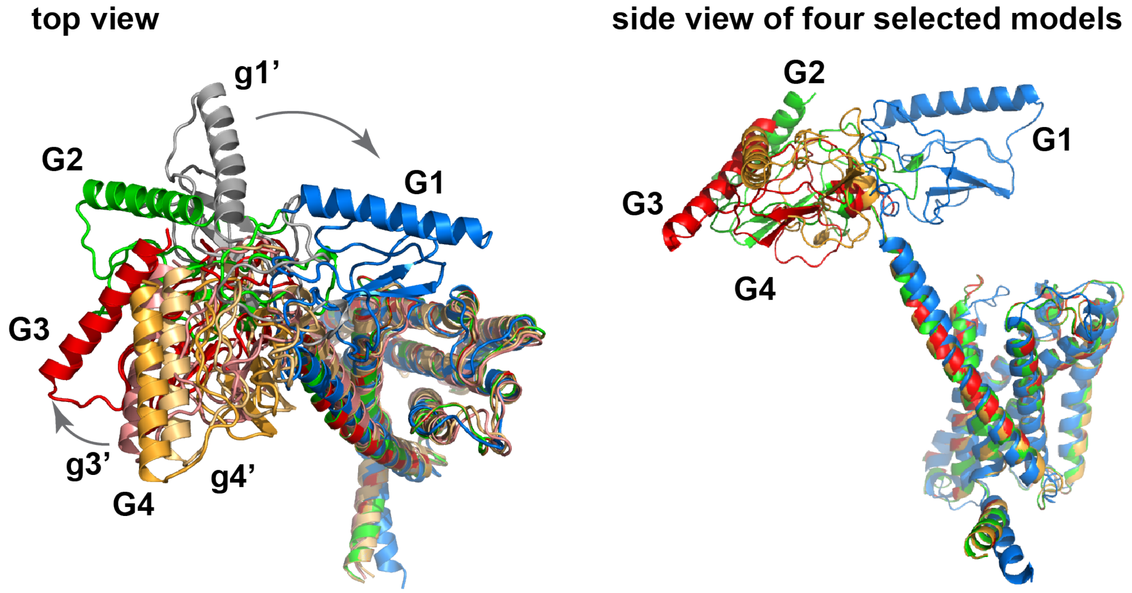

Supplementary Fig. 2 Top view and side view of models generated from rotating ECD of Model 2 (Supplementary Fig. S1). Extended simulations were only carried out from the starting models of G1, G2, G3, and G4. We did not pursue the simulations starting from the models of g1', g3', and g4', since they were converted to similar intermediate conformations of G1, G3, and G4 after 36, 5, and 2 ns.

The Wootten numbering. Similar to the Ballesteros–Weinstein numbering scheme[5] for class A GPCRs, the Wootten numbering scheme[6] helps to identify the conserved residues in the transmembrane (TM) helices of class B GPCRs and facilitates the comparisons among members in the same class. The most conserved residue in each TM helix is designated X.50b (the "b" after the number is to identify class B when comparing with class A), where X is the TM helix number and all other residues in that helix are numbered relative to this conserved position (highlighted in bold in Supplementary Table 1).

The gridcount method. Gridcount[7] is an analysis tool that creates 3D (number) densities from molecular dynamics trajectories. It is used to look at the density of water or ions near proteins or in channels and pores. First, it counts occurrences of molecules on a 3D grid over a MD trajectory, and then creates (after proper normalization) a 3D density map. Next, the map is calculated by the cylindrical and slice averages and converted into a density map that is readable by VMD.[8]

Supplementary Table 1. Transmembrane domain sequence alignment of PAC1R with GCGR and CRF1R in the Wootten numbering[6].

		42	43	44	45	46	47	48	49	50	51	52	53	54	55	56	57	58	59	60	61	62	63	64
		156	157	158	159	160	161	162	163	**164**	165	166	167	168	169	170	171	172	173	174	175	176	177	178
TM1	PAC1R	L	Y	T	V	G	Y	S	T	**S**	L	V	T	L	T	T	A	M	V	I	L	C	R	F
		144	145	146	147	148	149	150	151	**152**	153	154	155	156	157	158	159	160	161	162	163	164	165	166
	GCGR	M	Y	T	V	G	Y	S	L	**S**	L	G	A	L	L	L	A	L	A	I	L	G	G	L
		122	123	124	125	126	127	128	129	**130**	131	132	133	134	135	136	137	138	139	140	141	142	143	144
	CRF1R	I	N	Y	L	G	H	C	I	**S**	L	V	A	L	L	V	A	F	V	L	F	L	R	A

		48	49	50	51	52	53	54	55	56	57	58	59	60	61	62	63	64	65	66
		187	188	**189**	190	191	192	193	194	195	196	197	198	199	200	201	202	203	204	205
TM2	PAC1R	F	I	**H**	M	N	L	F	V	S	F	M	L	R	A	I	S	V	F	I
		175	176	**177**	178	179	180	181	182	183	184	185	186	187	188	189	190	191	192	193
	GCGR	A	I	**H**	A	N	L	F	A	S	F	V	L	K	A	S	S	V	L	V
		153	154	**155**	156	157	158	159	160	161	162	163	164	165	166	167	168	169	170	171
	CRF1R	I	I	**H**	A	N	L	I	A	A	F	I	L	R	N	A	T	W	F	V

		31	32	33	34	35	36	37	38	39	40	41	42	43	44	45	46	47	48	49	50	51	52	53	54	55	56
		228	229	230	231	232	233	234	235	236	237	238	239	240	241	242	243	244	245	246	**247**	248	249	250	251	252	253
TM3	PAC1R	A	V	M	V	F	F	H	Y	C	V	V	S	N	Y	F	W	L	F	I	**E**	G	L	Y	L	F	T
		226	227	228	229	230	231	232	233	234	235	236	237	238	239	240	241	242	243	244	**245**	246	247	248	249	250	251
	GCGR	V	A	A	V	F	M	Q	Y	G	I	V	A	N	Y	C	W	L	L	V	**E**	G	L	Y	L	H	N
		190	191	192	193	194	195	196	197	198	199	200	201	202	203	204	205	206	207	208	**209**	210	211	212	213	214	215
	CRF1R	L	V	T	A	A	Y	N	Y	F	H	V	T	N	F	F	W	M	F	G	**E**	G	C	Y	L	H	T

		45	46	47	48	49	50	51	52	53	54	55	56	57	58	59	60	61	62	63	64	65	66	67
		269	270	271	272	273	**274**	275	276	277	278	279	280	281	282	283	284	285	286	287	288	289	290	291
TM4	PAC1R	Y	T	I	I	G	**W**	G	T	P	T	V	C	V	T	V	W	A	T	L	R	L	Y	F
		267	268	269	270	271	**272**	273	274	275	276	277	278	279	280	281	282	283	284	285	286	287	288	289
	GCGR	Y	L	G	I	G	**W**	G	A	P	M	L	F	V	V	P	W	A	V	V	K	C	L	F
		231	232	233	234	235	**236**	237	238	239	240	241	242	243	244	245	246	247	248	249	250	251	252	253
	CRF1R	F	I	C	I	G	**W**	G	V	P	F	P	I	I	V	A	W	A	I	G	K	L	Y	Y

		40	41	42	43	44	45	46	47	48	49	50	51	52	53	54	55	56	57	58	59	60	61	62
		310	311	312	313	314	315	316	317	318	319	**320**	321	322	323	324	325	326	327	328	329	330	331	332
TM5	PAC1R	K	G	P	V	V	G	S	I	M	V	**N**	F	V	L	F	I	G	I	I	V	I	L	V
		308	309	310	311	312	313	314	315	316	317	**318**	319	320	321	322	323	324	325	326	327	328	329	330
	GCGR	R	F	P	V	F	L	A	I	L	I	**N**	F	F	I	F	V	R	I	V	Q	L	L	V
		273	274	275	276	277	278	279	280	281	282	**283**	284	285	286	287	288	289	290	291	292	293	294	295
	CRF1R	Q	G	P	M	A	L	V	L	L	I	**N**	F	I	F	L	F	N	I	V	R	I	L	M

		38	39	40	41	42	43	44	45	46	47	48	49	50	51	52	53	54	55	56	57	58
		351	352	353	354	355	356	357	358	359	360	361	362	**363**	364	365	366	367	368	369	370	371
TM6	PAC1R	L	A	R	S	T	L	L	L	I	P	L	F	**G**	I	H	Y	T	V	F	A	F
		347	348	349	350	351	352	353	354	355	356	357	358	**359**	360	361	362	363	364	365	366	367
	GCGR	L	A	K	S	T	L	T	L	I	P	L	L	**G**	V	H	E	V	V	F	A	F
		312	313	314	315	316	317	318	319	320	321	322	323	**324**	325	326	327	328	329	330	331	332
	CRF1R	A	V	K	A	T	L	V	L	L	P	L	L	**G**	I	T	Y	M	L	A	F	V

		43	44	45	46	47	48	49	50	51	52	53	54	55	56	57	58	59	60	61	62
		386	387	388	389	390	391	392	**393**	394	395	396	397	398	399	400	401	402	403	404	405
TM7	PAC1R	L	G	L	G	S	F	Q	**G**	F	V	V	A	V	L	Y	C	F	L	N	G
		386	387	388	389	390	391	392	**393**	394	395	396	397	398	399	400	401	402			
	GCGR	L	F	L	S	S	F	Q	**G**	L	L	V	A	V	L	Y	C	F			
		349	350	351	352	353	354	355	**356**	357	358	359	360	361	362	363	364	365	366	367	368
	CRF1R	A	F	L	E	S	F	Q	**G**	F	F	V	S	V	F	A	C	F	L	N	S

Note: The conserved residues in TM1-7 are highlighted in bold.

Supplementary Information B: The construction, validation, and analyses of our Markov state model (MSM)

We used the MSMBuilder[9,10] program to construct the MSM transition matrix between the open and closed conformations and calculated the transition timescale based on the transition-path theory.[11-14] We first prepared the trajectory dataset with atoms indices by saving the C_α coordinates of res. 30-419. The C_α atoms are often used to represent the overall protein structures and the dynamic terminal residues should be excluded. With the structural stabilities of our PAC1R models in the microsecond MD refinements (by RMSD measurement in Supplementary Fig. 5), each PAC1R model reached to a relatively stable state after 200~500 ns, which had been continuously relaxed to prove the stability for another 1.5~2.5 μs. The percentages of the open and closed states were 23.2%, 17.5%, 19.9%, and 25.1% for G4, G1, G2, and G3 respectively in the total conformations we sampled. Therefore, we have collected six shorter MD simulations of 20~50 ns each, and the first 200~550 ns from the long simulations, totaling 6,324 configurations.

Next, we grouped the conformation dataset into a set of clusters, called microstates, based on structural similarities. We used the *k*-centers algorithm[9] to group the dataset into 55 clusters by RMSD metric with mean distance of ~0.34 Å and maximum distance of ~0.56 Å, which is in the range of the RMSD standard deviations of the last ~1.5 μs in Supplementary Fig. 3. The small cluster number, in a difference from the hundreds to thousands of intrinsic conformations of previous protein folding/unfolding studies,[12,15] is the result of limited conformational changes due to the linker rotation and partial melting.

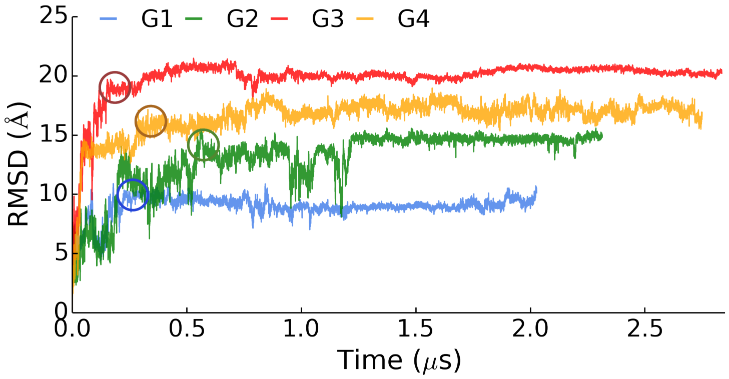

Supplementary Fig. 3 Time evolution of PAC1R RMSDs. RMSDs were computed by backbone alignments on initial structures with standard deviations of 0.34~0.56 Å in the last ~1.5 μs. Each PAC1R model reached to a relatively stable state after 200~500 ns, which had been continuously relaxed to prove the stability for another 1.5~2.5 μs. The conformational states between which we calculated the shortest pathways are circled.

With the set of microstates discretization, a series of microstate transition matrixes in the evolution of the observation interval or lag time at τ, 2τ, …, $n\tau$, were constructed, on which the implied timescales and the *Chapman-Kolmogorov* test[16] were carried out to examine if a microstate transition matrix with the microstate discretization and a chosen lag time is Markovian.[12,16,17] We used maximum likelihood estimation to build the transition matrixes at series of lag times, in which increasing the lag time means that states can get larger and more coarse grained as the longer lag time the fewer states are kinetically relevant (kinetically reach each other on timescales faster than the lag time).[18] The implied timescales as a function of the lag time and the eigenvalues of the transition matrix are shown in in Supplementary Fig. 4. The macrostates number of four is determined by the number of the major gaps of the implied timescales as well as the number of eigenvalues of the transition matrix that are close to 1.[19] The division of four macrostates were calculated from the eigenfunction structure using the *Perron Cluster Cluster Analysis* (PCCA) method.[19] Consistently, the conformations of G1, G2, G3, and G4 were lumped into the four macrostates, A, B, C, and D, respectively.

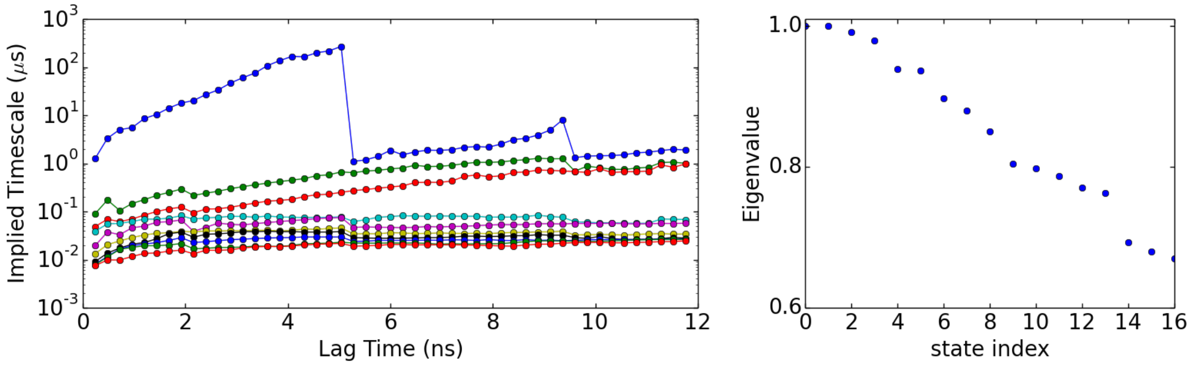

Supplementary Fig. 4 Left panel: Implied timescales as a function of the lag time. The macrostate partitions were robust within ranges of lag time series. Right panel: Eigenvalues of the transition matrix at lag time of 4.8 ns. Only the first seventeen data are shown.

The *Chapman-Kolmogorov* test can be implemented on an individual state or set of states.[12,16] In general, we compared the transition probability matrix $\mathbf{T}(n\tau)$ based on the transition counts (known as observed trajectory) and $[\mathbf{T}(\tau)]^n$ from MSM for a given set of states A defined at the starting microstate discretization.[12,16] The initial stationary distribution at time τ restricted to a set A is given by

$$w_i^A = \begin{cases} \dfrac{\pi_i}{\sum_{j\in A}\pi_j} & i \in A \\ 0 & i \notin A \end{cases}, \qquad (s-1)$$

where π is the stationary probability of the $m \times m$ transition matrix $\mathbf{T}(\tau)$. The trajectory-based time-dependence of the probability to be after time $n\tau$ with starting distribution $\mathbf{w}^A$ is given by

$$p_{\mathrm{MD}}(A, A;\ n\tau) = \sum_{i \in A} w_i^A\, p_{\mathrm{MD}}(i, A;\ n\tau), \qquad (s-2)$$

where $p_{\mathrm{MD}}(i, A;\ n\tau)$ is the trajectory-based estimate of the stochastic transition function given by

$$p_{\mathrm{MD}}(i, A;\ n\tau) = \frac{\sum_{j \in A} c_{ij}^{\mathrm{obs}}(n\tau)}{\sum_{j=1}^{m} c_{ij}^{\mathrm{obs}}(n\tau)}, \qquad (s-3)$$

where $c_{ij}^{\mathrm{obs}}(n\tau)$ is number of transition counts between states *i* and *j* at time $n\tau$.

Likewise, the probability to be at *A by* Markov model is given by

$$p_{\mathrm{MSM}}(A, A;\ n\tau) = \sum_{i \in A} [(\mathbf{w}^A)^T\ \mathbf{T}^n(\tau)]_i\ , \qquad (s-4)$$

We tested how well the equality $p_{\mathrm{MD}}(A, A;\ n\tau) = p_{\mathrm{MSM}}(A, A;\ n\tau)$ holds, as whether the solid line is within the error bar range of the dash line in Supplementary Fig. 5. The uncertainties of the transition probabilities estimated from the MD trajectories are computed as:

$$\epsilon_{\mathrm{MD}}(A, A;\ n\tau) = \sqrt{n \frac{p_{\mathrm{MD}}(A, A;\ n\tau) - [p_{\mathrm{MD}}(A, A;\ n\tau)]^2}{\sum_{i \in A} \sum_{j=1}^{m} c_{ij}^{\mathrm{obs}}(n\tau)}}. \qquad (s-5)$$

There are around 27 microstates that constitute the shortest and second shortest transition pathways between closed and open states, and were identified as four subsets based on the macrostate division. The *Chapman-Kolmogorov* test of the four subsets is shown in Supplementary Fig. 5, all of which hold the test within the statistical uncertainty. The transition probabilities from MSM agree well with the probabilities in observed trajectory within statistical uncertainty at lag time of 1.68 ns. Thus, we built up a four macrostates MSM with 94% data in use with lag time of 1.68 ns.

Using the transition-path theory (TPT),[11-14] we calculated the minimum transition net flux of the shortest pathway connecting the closed states or from the open states to closed states in the transition matrix. With the lag time divided by the minimum transition net flux, we obtained the time to travel from one set of states to the other. The minimum transition net flux, number of microstates, and estimated transition time in the shortest pathway between closed and open states are summarized in Supplementary Table 2. The pathways connecting the closed states (G1, G2, and G3) are relatively short, but from closed states to the open states (G4) it is rather remote, suggesting a clear partition in the conformational states. Regarding the minimum transition flux, the reversible transitions within the

closed states are about 10-30 times faster than the transition from open states to a closed one. Key conformations in the shortest pathways among closed and open states are summarized in Fig. 1A.

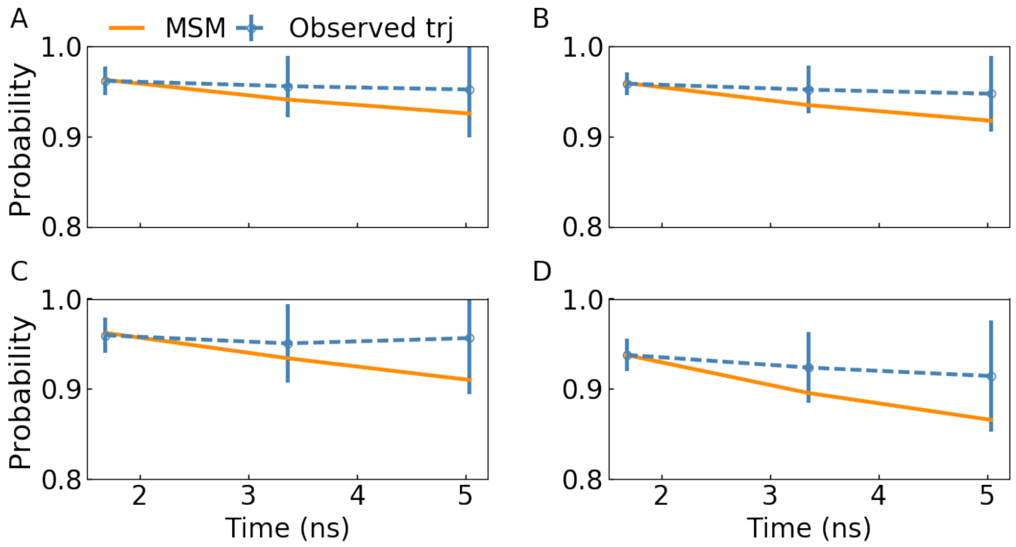

Supplementary Fig. 5 Microstates that constitute the shortest and second shortest transition pathways between closed and open states were divided into four subsets (A, B, C, and D) according to macrostate division and examined by Chapman-Kolmogorov test. The transition probabilities from MSM agree well with the probabilities in the observed trajectories within statistical uncertainty.

Supplementary Table 2. The minimum transition net flux, the number of microstates, and the estimated transition time of the shortest pathways between closed and open states shown in Fig. 1A.

	G1-G2	G1-G3	G2-G3	G1-G4	G2-G4	G3-G4
Minimum net flux (10^{-5})	6.10	7.17	7.84	0.41	0.26	0.59
Number of microstates	3	5	3	13	12	11
Transition time (μs)	27.6	23.4	21.4	412.3	650	284.5

Note: G1, G2, G3 and G4 are represented by one or two microstates.

Supplementary Information C: Details of the PAC1R orientations and conformations

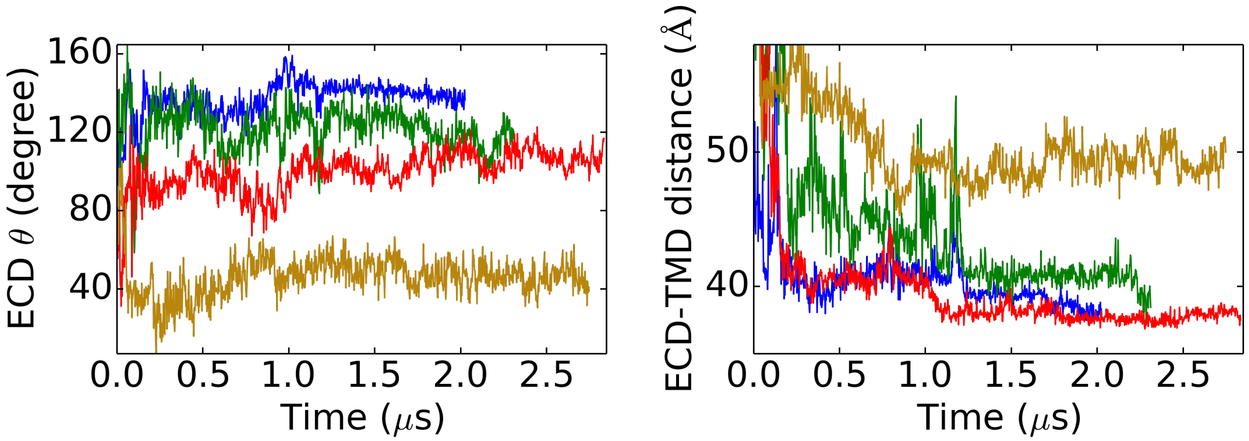

Supplementary Fig. 6 Time evolutions of the ECD orientation (θ) and the ECM-7TM separation in G1-G4 trajectories. After 0.2-1.2 μs, three of four simulations (G1-G3) turned out to form close ECD-7TM contacts — as shown by the ECD-7TM distance mostly within 42 Å. The open conformation of the PAC1R was stable and lasted for over 1.5 μs to the end of the simulation. The color scheme is consistent with Fig. 1.

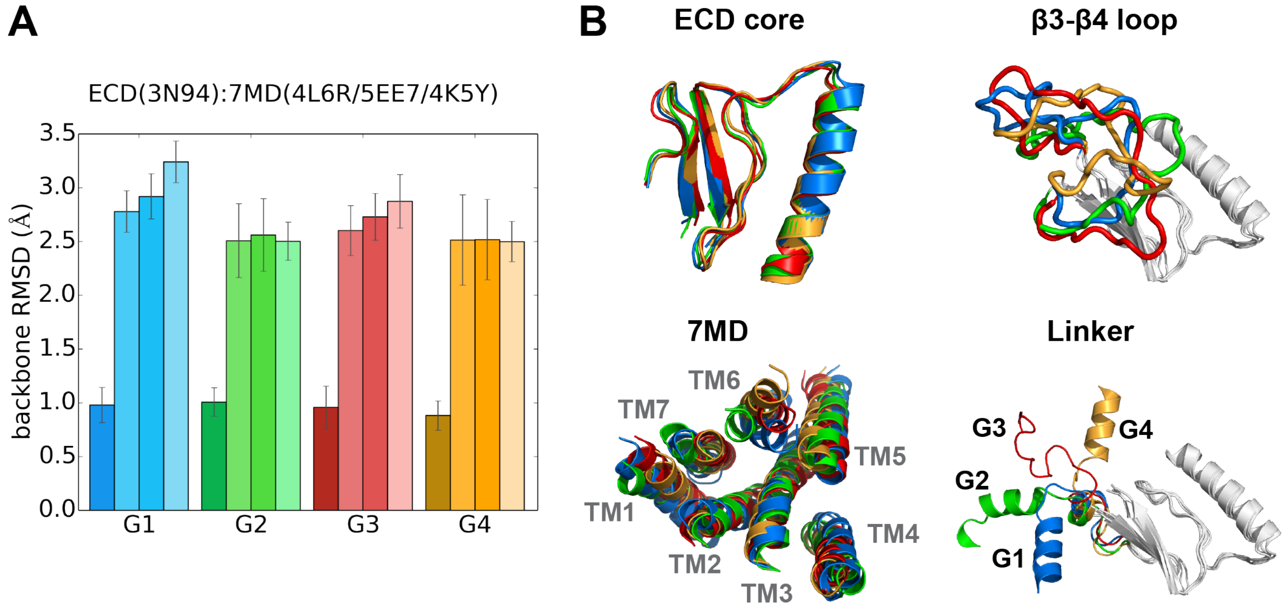

Supplementary Fig. 7 Structural stability of the PAC1R models after microsecond MD refinements. **(A)** RMSDs with standard errors (± 0.4 Å) computed by backbone alignments of ECD core on crystal structure (PDB ID: 3N94), and 7TM on GCGR (PDB ID: 4L6R and 5EE7) and CRF1R (PDB ID: 4K5Y) for four ligand-free states (G1-G4). **(B)** Sideview of the ECD core alignments, topview of the 7TM alignment, and sideviews of the β3-β4 loop and linker of the four final states are shown on the right. The color scheme and line types are consistent with Fig. 1.

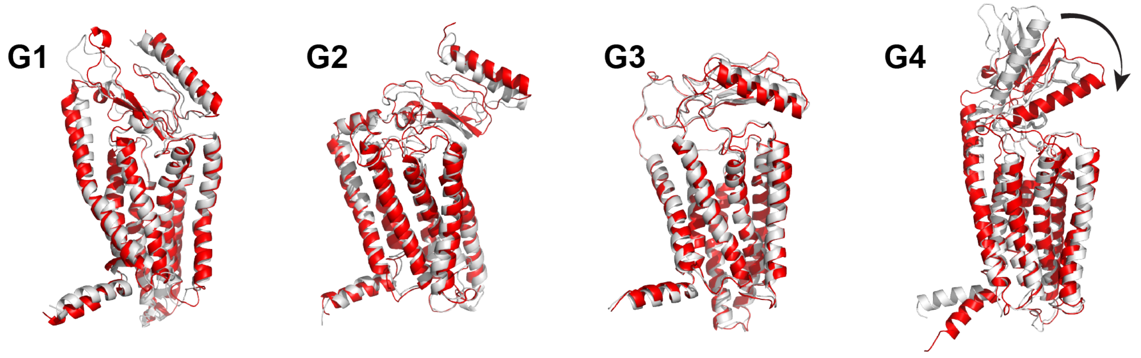

Supplementary Fig. 8 Overlapped cartoon illustrations of the initial (white) and final (red) conformations in four PAC1R-short model simulations with res. 89-109 deleted from the final models of our PAC1R simulations; each system was simulated for 120~160 ns.

Supplementary Information D: Helices distance and Residue-residue minimum-distance maps

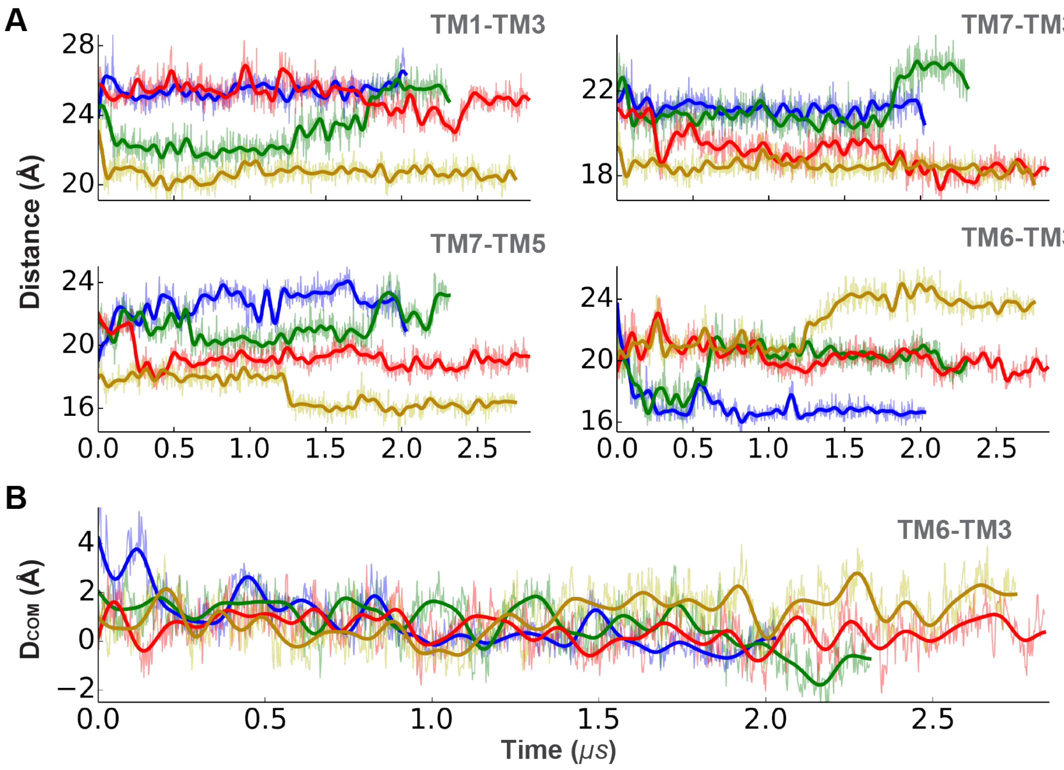

Supplementary Fig. 9 (A) Plots of the helical separation changing over time. **(B)** Plot of TM6 Z-COM shift over time.

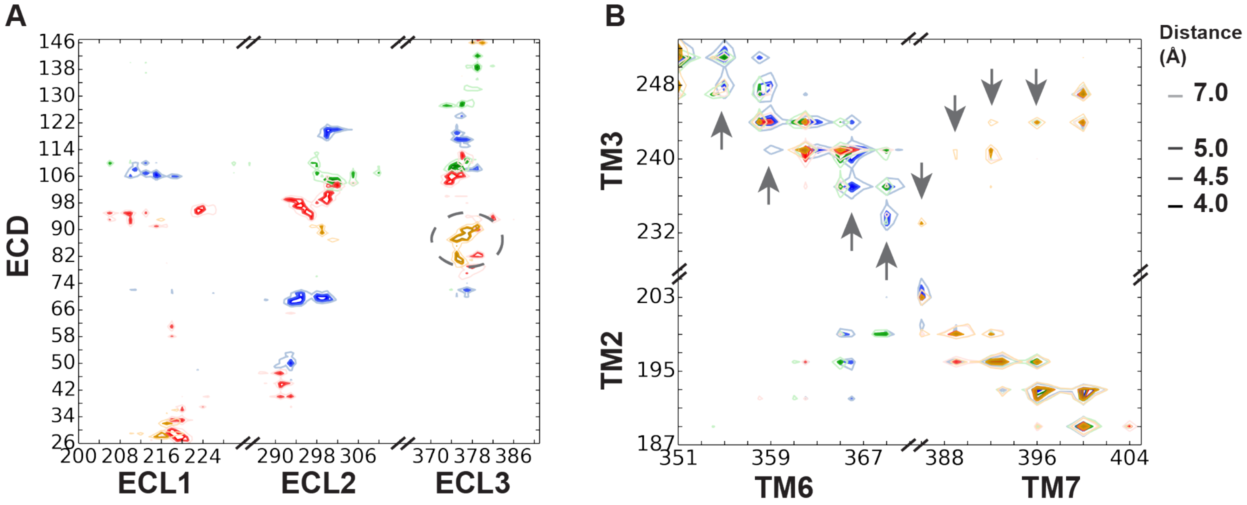

Supplementary Fig. 10 The residue-residue minimum-distance maps of **(A)** ECD and ECLs and of **(B)** TM2-TM3 and TM6-TM7. Data were collected and averaged from the last 100 ns of each trajectory. In (B), the upward-pointing arrows represent the residue pair getting closer and the downward-pointing arrows represent the residue pair getting father in the open-to-closed transition. The color scheme is consistent with Fig. 1.

Supplementary Information E: PACAP-bound models in the open and closed states

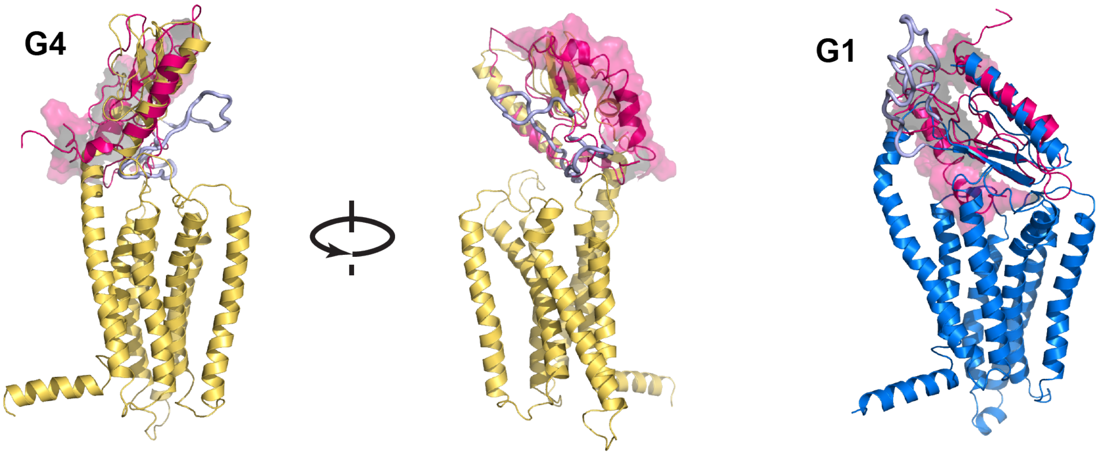

Supplementary Fig. 11 Superposition of the PAC1R ECD-PACAP$_{6\text{-}38}$ solution structure (PDB ID: 2JOD, purple) on our open-state (gold) and closed-state final models (blue). Our open-state model (G4) clearly shows that the 21-aa sequence does not impact the peptide-binding groove,[20] which provides an explanation to the unaltered binding properties from 21-aa deletion in previous studies.[21]

References

1 Biasini, M. *et al.* SWISS-MODEL: modelling protein tertiary and quaternary structure using evolutionary information. *Nucleic Acids Res.* **42**, W252-W258 (2014).

2 Siu, F. Y. *et al.* Structure of the human glucagon class B G-protein-coupled receptor. *Nature* **499**, 444-449 (2013).

3 Hollenstein, K. *et al.* Structure of class B GPCR corticotropin-releasing factor receptor 1. *Nature* **499**, 438-443 (2013).

4 Kumar, S., Pioszak, A., Zhang, C., Swaminathan, K. & Xu, H. E. Crystal Structure of the PAC1R Extracellular Domain Unifies a Consensus Fold for Hormone Recognition by Class B G-Protein Coupled Receptors. *PLoS ONE* **6**, e19682 (2011).

5 Ballesteros, J. A. & Weinstein, H. in *Methods in Neurosciences* Vol. Volume 25 (ed C. Sealfon Stuart) 366-428 (Academic Press, 1995).

6 Wootten, D., Simms, J., Miller, L. J., Christopoulos, A. & Sexton, P. M. Polar transmembrane interactions drive formation of ligand-specific and signal pathway-biased family B G protein-coupled receptor conformations. *Proc. Natl. Acad. Sci. U. S. A.* **110**, 5211-5216 (2013).

7 Beckstein, O. & Sansom, M. S. P. Liquid-vapor oscillations of water in hydrophobic nanopores. *Proc. Natl. Acad. Sci. U. S. A.* **100**, 7063-7068 (2003).

8 Humphrey, W., Dalke, A. & Schulten, K. VMD: visual molecular dynamics. *J. Mol. Graph. Model.* **14**, 33-38 (1996).

9 Bowman, G. R., Huang, X. H. & Pande, V. S. Using generalized ensemble simulations and Markov state models to identify conformational states. *Methods* **49**, 197-201 (2009).

10 Bowman, G. R., Beauchamp, K. A., Boxer, G. & Pande, V. S. Progress and challenges in the automated construction of Markov state models for full protein systems. *J. Chem. Phys.* **131**, 124101 (2009).

11 E, W. & Vanden-Eijnden, E. Towards a theory of transition paths. *J. Stat. Phys.* **123**, 503-523 (2006).

12 Noe, F., Schutte, C., Vanden-Eijnden, E., Reich, L. & Weikl, T. R. Constructing the equilibrium ensemble of folding pathways from short off-equilibrium simulations. *Proc. Natl. Acad. Sci. U. S. A.* **106**, 19011-19016 (2009).

13 Berezhkovskii, A., Hummer, G. & Szabo, A. Reactive flux and folding pathways in network models of coarse-grained protein dynamics. *J. Chem. Phys.* **130**, 205102 (2009).

14 Metzner, P., Schutte, C. & Vanden-Eijnden, E. Transition Path Theory for Markov Jump Processes. *Multiscale Modeling & Simulation* **7**, 1192-1219 (2009).

15 Lane, T. J., Bowman, G. R., Beauchamp, K., Voelz, V. A. & Pande, V. S. Markov State Model Reveals Folding and Functional Dynamics in Ultra-Long MD Trajectories. *J. Am. Chem. Soc.* **133**, 18413-18419 (2011).

16 Prinz, J. H. *et al.* Markov models of molecular kinetics: Generation and validation. *J. Chem. Phys.* **134**, 174105 (2011).

17 Chodera, J. D., Singhal, N., Pande, V. S., Dill, K. A. & Swope, W. C. Automatic discovery of metastable states for the construction of Markov models of macromolecular conformational dynamics. *J. Chem. Phys.* **126**, 155101 (2007).

18 Pande, V. S., Beauchamp, K. & Bowman, G. R. Everything you wanted to know about Markov State Models but were afraid to ask. *Methods* **52**, 99-105 (2010).

19 Noe, F., Horenko, I., Schutte, C. & Smith, J. C. Hierarchical analysis of conformational dynamics in biomolecules: transition networks of metastable states. *J. Chem. Phys.* **126**, 155102 (2007).

20 Sun, C. H. *et al.* Solution structure and mutational analysis of pituitary adenylate cyclase-activating polypeptide binding to the extracellular domain of PAC1-Rs. *Proc. Natl. Acad. Sci. U. S. A.* **104**, 7875-7880 (2007).

21 Blechman, J. & Levkowitz, G. Alternative Splicing of the Pituitary Adenylate Cyclase-Activating Polypeptide Receptor PAC1: Mechanisms of Fine Tuning of Brain Activity. *Front. Endocrinol. (Lausanne)* **4**, 1 (2013).
